# Text Mining of Symptom Descriptions in the Vaccine Adverse Event Reporting System for Human Papillomavirus Vaccination

**DOI:** 10.7759/cureus.92575

**Published:** 2025-09-17

**Authors:** Kohei Shiota, Megumi Horibe, Yoko Ino, Mari Iwata, Hideyuki Tanaka, Mayumi Kitamura, Kazuhiro Iguchi, Mitsuhiro Nakamura

**Affiliations:** 1 Laboratory of Drug Informatics, Gifu Pharmaceutical University, Gifu, JPN; 2 Department of Nursing, School of Health Sciences, Asahi University, Gifu, JPN; 3 Laboratory of Pharmaceutical Health Care and Promotion, Gifu Pharmaceutical University, Gifu, JPN; 4 Department of Pharmacy, Yanaizu Pharmacy, Gifu, JPN; 5 Laboratory of Community Pharmacy, Gifu Pharmaceutical University, Gifu, JPN

**Keywords:** human papillomavirus vaccine, sentiment analysis, text mining, vaccine administration, vaccine adverse event reporting system

## Abstract

Introduction: The World Health Organizationhas reaffirmed the efficacy and safety of human papillomavirus (HPV) vaccines in a statement. While vaccine safety has been extensively studied, little is known about the descriptive language used in reports of adverse events. The Vaccine Adverse Event Reporting System (VAERS), managed by the US Food and Drug Administration and the Centers for Disease Control and Prevention, collects free-text narratives on adverse events following vaccination. This study aimed to examine these narratives to describe vocabulary patterns associated with HPV vaccination.

Methods: We conducted a retrospective, cross-sectional observational study using quantitative text mining techniques. Symptom descriptions related to HPV vaccination were extracted from the Vaccine Adverse Event Reporting System (VAERS, 2009-2023). Sentiment analysis was performed with the AFINN lexicon in R (“tidytext” package), which assigns numerical sentiment scores to words. This quantitative scoring approach enabled us to describe vocabulary patterns without qualitative inference of context or emotions.

Results: Sentiment analysis was performed using the R “tidytext” package with the AFINN lexicon. Reports on suspected adverse events were obtained from the VAERS reports spanning 2009 to 2023, comprising 55,919 suspected adverse events. Approximately 6% of these reports involved product handling issues. The analysis showed that sentiments toward vaccination were more negative among females than males.

Conclusion: Healthcare providers supporting HPV vaccination should provide patients with accurate and comprehensive information on vaccine safety and potential adverse reactions.

## Introduction

The peak incidence of cervical cancer occurs during a woman’s most fertile years, typically between her late 20s and early 40s. Late detection of cervical cancer can lead to fertility loss, significantly impacting reproductive health. Cervical cancer is primarily caused by human papillomavirus (HPV), and it is estimated that most sexually active individuals will be infected with HPV at least once in their lifetime [1-5]. HPV is the most common sexually transmitted disease in the United States. Approximately 10% of men and 3.6% of women are infected with oral HPV, which is believed to cause 60-70% of oropharyngeal cancers in the United States [6].

Cervical and hepatic cancers are reportedly the only cancers that can be prevented. A dual prevention strategy involving HPV vaccination (primary prevention) and cervical cancer screening (secondary prevention) has been established. Early detection and treatment of cervical cancer result in a five-year relative survival rate of 76.5%. Adverse reactions to HPV vaccination in the United States (US) from 2006 to 2008 were reported at a rate of 53.9 per 100,000 doses distributed, with only 3.3 reports of serious adverse reactions per 100,000 doses distributed [7]. The World Health Organization [8] reaffirmed the efficacy and safety of HPV vaccines in a 2015 statement.

The Vaccine Adverse Event Reporting System (VAERS) is a US vaccine safety surveillance database managed by the Food and Drug Administration (FDA) and the Centers for Disease Control and Prevention (CDC) [9-13]. VAERS collects reports of suspected adverse events (SAE) across all age groups after the administration of any US-licensed vaccine. The VAERS database contains both events that occur by chance and those causally related to vaccination, as distinguishing between the two can be challenging. While vaccine safety has been extensively studied, research on patients’ emotional responses to vaccination remains limited. Although the efficacy and safety of the HPV vaccine are well established, little is known about how recipients’ subjective experiences are reflected in post-vaccination reports. To address this gap, we analyzed free-text narratives from VAERS, where reporters freely enter detailed symptom descriptions. While VAERS does not directly solicit emotional responses, symptom narratives may contain linguistic elements that reflect psychological or emotional stress associated with vaccination. In this study, we examined these textual data to describe linguistic patterns in symptom narratives related to HPV vaccination, with particular attention to expressions that may indirectly reflect psychological or emotional stress.

## Materials and methods

Data collection

The present study was designed as a retrospective, cross-sectional observational study, utilizing anonymized data from the VAERS. Data for this study were obtained from the VAERS database, managed by the US Department of Health and Human Services [9]. The VAERS database accepts reports from multiple sources, including healthcare professionals, vaccine recipients, and their guardians. These reports are processed and maintained by the FDA and the CDC. All data were fully anonymized by the regulatory authorities. Data on SAEs related to HPV vaccines were extracted from the VAERS databases. SAEs were defined in accordance with the Medical Dictionary for Regulatory Activities version 27.0 [14]. In the VAERS database, the SAE data for each case are presented as three file sets: VAERSDATA.CSV, VAERSVAX.CSV, and VAERSSYMPTOMS.CSV [10]. These data were integrated into a relational database using FileMaker Pro Advanced 17 (FileMaker Inc., Santa Clara, California, USA).

Sentiment analysis

**Figure 1:**
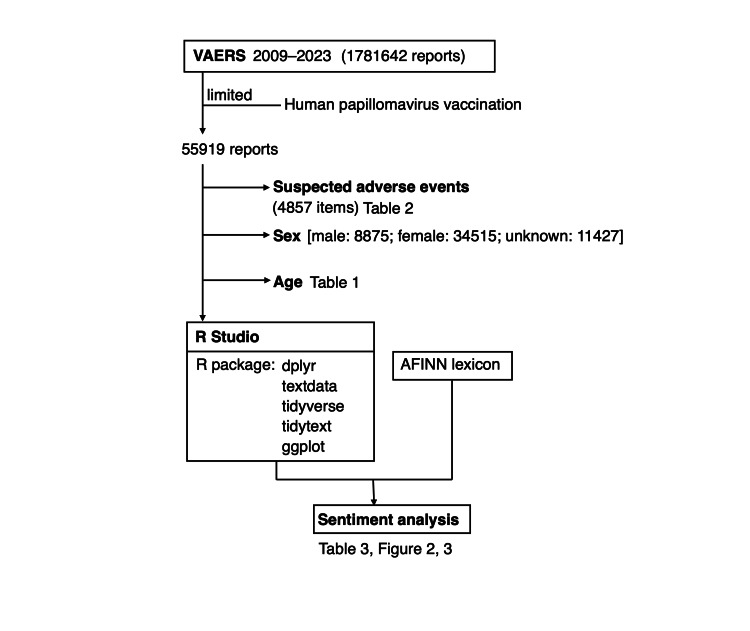
Flowchart of the text mining analysis

We adopted a quantitative text mining approach, focusing on word-level frequency and sentiment scoring rather than qualitative interpretation of narratives (Figure 1). To determine the expressed tones in each free-text description, we used the AFINN lexicon [15], developed by Finn Arup Nielsen. The AFINN lexicon assigns a score ranging from -5 to + 5 to each word, with negative scores suggesting negative sentiment. The overall sentiment of each text was determined by summing the sentiment scores of all words in the text. The analysis was conducted using R packages, including “tidytext,” “dplyr,” “textdata,” “tidyverse,” and “ggplot” [16] to access the lexicon and process the data for sentiment scoring. Data analysis was performed using RStudio 2024.09.1 (Posit, Boston, Massachusetts, USA).

As a supplementary analysis, we also applied the National Research Council (NRC) dictionary, which classifies words into two sentiments (positive and negative) and eight emotions (anger, anticipation, disgust, fear, joy, sadness, surprise, and trust) [17]. This supplementary analysis was conducted to provide additional support for the main findings. 

Ethical considerations 

Ethical approval was not sought for this study because it is a retrospective, cross-sectional observational study without any research participants. The VAERS database is completely anonymized by the administrative authorities of the United States and is available to the public. Therefore, these data sources are not subject to Ethical Guidelines for Medical Research Involving Human Subjects (Ethical Guidelines for Medical Science). 

## Results

Data from 2009 to 2023 were obtained from the VAERS database, comprising 55,919 SAEs across 4,857 items. Regarding age distribution, cases were primarily concentrated among teenagers and young adults in their 20s in the US, corresponding to the recommended age for HPV vaccination (Table 1).

**Table 1:** Reporting rate of suspected adverse events for human papillomavirus vaccine ^*^Percentage of the number of reports for each age group in the total number of HPV vaccine reports.

Age (years)	Reports (total)	HPV vaccine reports (n)	%^*^
Total	1065472	38239	-
<10	224669	939	2.5
10–19	139664	29175	76.3
20–29	133304	7037	18.4
30–39	179377	657	1.7
40–49	179718	330	0.9
50–59	208740	101	0.3

Among vaccine types, HPV4 was the most frequently reported in the VAERS database, accounting for approximately 66.5% of cases. Reporting rates in the VAERS database for HPV9, HPV2, and HPVX were 31.2%, 0.46%, and 1.88%, respectively. Commonly reported symptoms in VAERS included dizziness, syncope, headache, and nausea, as detailed in Table 2. Additionally, approximately 6% of reported cases in the VAERS database were related to product handling issues, such as improper product storage and incorrect dosing schedules (Table 2).

**Table 2:** Number of reports for the top 40 events associated with human papillomavirus vaccination in the vaccine adverse event reporting system No adverse event (n = 12563).

Adverse events	Number of reports (n)	Rate (%)
Dizziness	5642	3.11
Syncope	5621	3.10
Incorrect product storage	4983	2.75
Headache	3884	2.14
Nausea	3577	1.97
Inappropriate schedule of drug administration	2849	1.57
Injection site pain	2670	1.47
Loss of consciousness	2664	1.47
Pyrexia	2589	1.43
Pain	2252	1.24
Vomiting	2174	1.20
Fatigue	2165	1.19
Injection site erythema	2151	1.19
Pallor	2119	1.17
Pain in extremity	2021	1.11
Injection site swelling	1899	1.05
Urticaria	1798	0.99
Rash	1784	0.98
Product storage error	1680	0.93
Erythema	1640	0.90
Immediate post-injection reaction	1520	0.84
Incorrect storage of drug	1381	0.76
Fall	1376	0.76
Drug exposure during pregnancy	1367	0.75
Asthenia	1257	0.69
Hypoaesthesia	1182	0.65
Arthralgia	1179	0.65
Pruritus	1136	0.63
Paraesthesia	1111	0.61
Convulsion	1090	0.60
Dyspnoea	1069	0.59
Malaise	1041	0.57
Hyperhidrosis	984	0.54
Tremor	936	0.52
Injection site warmth	851	0.47
Myalgia	838	0.46
Inappropriate schedule of product administration	827	0.46
Head injury	750	0.41
Abdominal pain	748	0.41
Exposure during pregnancy	747	0.41

For males, we identified 304752, 190167, and 51417 words in reports related to the 10-14, 15-19, and 20-44 year age groups, respectively. The corresponding word counts for females were 922144, 1161891, and 879350, respectively. The mean sentiment scores based on the AFINN lexicon for males aged 10-14, 15-19, and 20-44 years were -0.6882962, -0.7186481, and -0.8455512, respectively. For females, the mean sentiment scores for the same age groups were -0.9130449, -0.8902707, and -0.9210978, respectively (Table 3).

**Table 3:** Mean AFINN score ^*^Matching words in the AFINN dictionary refer to words from the text that were assigned sentiment scores.

	Age (years)	Total word count	Matching word count^*^	Positive words^*^	Negative words^*^	Total score	Mean AFINN Score
Male	10–14	304752	10048	3350	6698	-6916	-0.6882962
	15–19	190167	5829	1865	3964	-4189	-0.7186481
	20–44	51417	1787	520	1267	-1511	-0.8455512
Female	10–14	922144	30027	8200	21827	-27416	-0.9130449
	15–19	1161891	32726	9096	23630	-29135	-0.8902707
	20–44	879350	22116	6000	16116	-20371	-0.9210978

**Figure 2:**
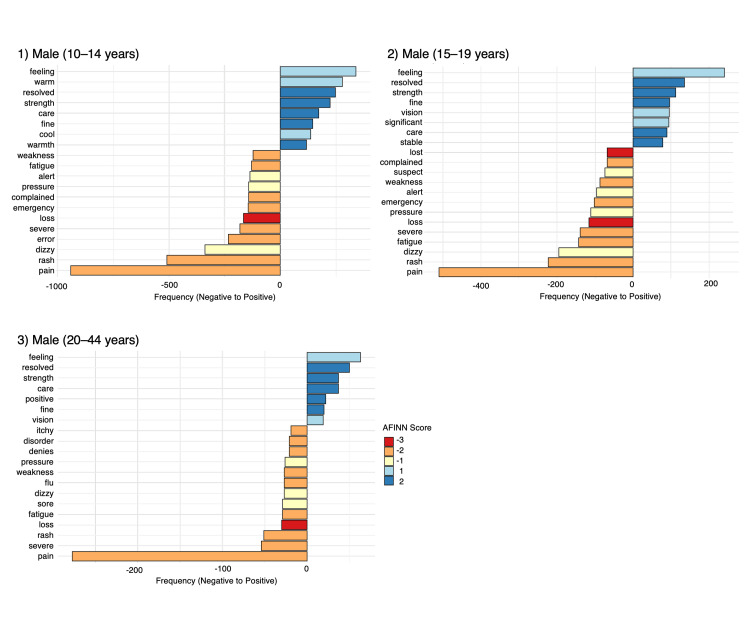
AFINN lexicon–graded positive and negative words in symptom texts of male VAERS reports The image is created by the author.

**Figure 3:**
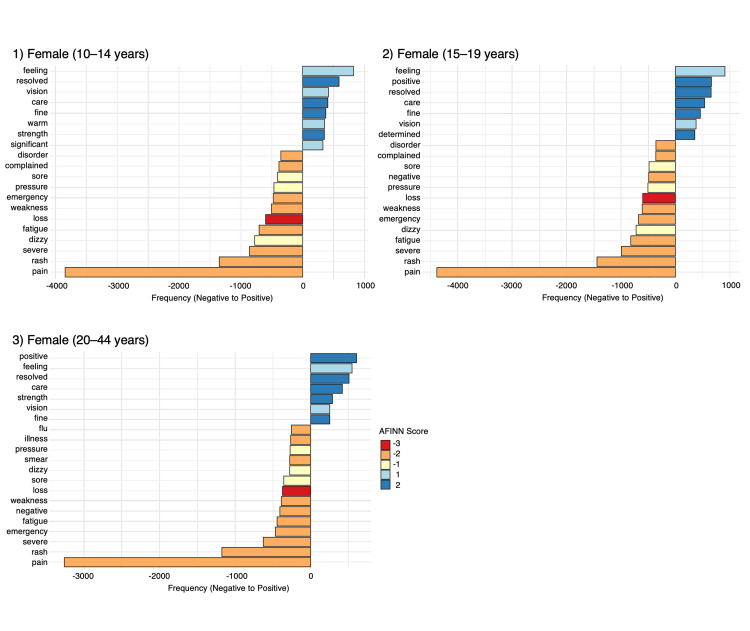
AFINN lexicon–graded positive and negative words in symptom texts of female VAERS reports The image is created by the author.

The top 20 words with AFINN sentiment scores for males and females in the 10-14, 15-19, and 20-44 years age groups are summarized in Figures 2, 3. Words such as “feeling,” “resolved,” “positive,” “care,” and “fine” were frequently used to express positive sentiments. In contrast, words like “pain,” “rash,” “severe,” “dizzy,” and “fatigue” were commonly used to convey negative sentiments. For males, the word “feeling” had the highest frequency among positive terms, while “resolved” and “strength” were the words with the highest AFINN sentiment scores. For females, the words “feeling” and “positive” had the highest frequencies among positive terms, while words such as “resolved” and “positive” had the highest AFINN sentiment scores. For both males and females, the word “pain” had the highest frequency among negative terms, while “loss” had the lowest AFINN sentiment score. The supplementary analysis using the NRC dictionary showed no effects of gender or age on the occurrence of words associated with these emotions (data not shown).

## Discussion

Although the efficacy and safety of the HPV vaccine are widely accepted, previous literature has noted that patients and their families seek information regarding vaccine effectiveness, potential adverse reactions, and long-term safety [8,18], as well as its limitations [19,20]. Therefore, healthcare providers are advised to provide accurate information and promote public information literacy to help distinguish between serious SAEs and common vaccination reactions.

In this study, we proposed using sentiment analysis to analyze emotional vocabulary in the VAERS free-text data. The VAERS data revealed that females expressed more negative sentiments than males, based on sentiment scores derived from the AFINN lexicon. The World Health Organization (WHO) introduced the concept of immunization stress-related response (ISRR), which includes acute reactions such as tachycardia, shortness of breath, dry mouth, limb numbness, dizziness, hyperventilation, and fainting. These reactions are often observed before, during, or immediately after vaccination. Physiological factors, such as those associated with adolescents and females, could contribute to these stress responses. The increased use of negative words by females may be linked to their higher likelihood of experiencing vasovagal reactions [21]. Additionally, both males and females in older age groups used more negative words. These results should be interpreted strictly as the frequency distribution of vocabulary in VAERS narratives, without inferring the precise context or subjective feelings of the reporters.

The reporting patterns associated with vaccines and vaccine lots were also analyzed. Approximately 85-90% of vaccine SAE reports involved relatively minor events, such as fever, redness, and swelling at the injection site [9]. The remaining <15% described serious events, including hospitalizations, life-threatening conditions, or deaths [9]. These reported serious events are of utmost concern, and it is possible that females are more concerned about such SAEs than males. 

From the current AFINN sentiment scores, positive words such as “resolved” and “strength”, as well as the negative word “loss”, are noteworthy. In interpreting these results, it is important to note the challenge of capturing contextual meaning through text mining. To supplement our sentiment analysis, we performed co-occurrence analysis of words within the same sentence. The results showed that the top four words co-occurring with “resolved” were outcome, recovered, reported, and symptoms. The words co-occurring with “strength” included dose, Gardasil, date, and lot. The words co-occurring with “loss” were patient, consciousness, hair, and weight. These patterns should be interpreted cautiously as linguistic associations rather than definitive contextual meanings (data not shown). Analyzing the contexts in which these words are used remains a subject for future investigation.

It should be noted that VAERS is primarily designed to capture symptom descriptions of suspected adverse events, rather than patients’ emotions or psychological states. Consequently, the emotional content identified in our sentiment analysis arises indirectly, through the language used to describe symptoms and experiences. For instance, words such as ‘fear,’ ‘loss,’ or ‘fatigue’ may not only denote physical or clinical conditions but may also reflect underlying psychological stress. Therefore, our interpretation of sentiment should be understood as an indirect proxy of emotional responses, derived from symptom narratives rather than from explicit self-reported feelings.

Analyzing the psychological basis of emotional vocabulary provides valuable insights into the emotional experiences of illness from the patient’s perspective, helping guide psychosocial interventions [22-24]. This study used the AFINN lexicon, which assigns words a score ranging from -5 to 5 according to the associated sentiment, because it allows for group comparisons. In recent years, intervention strategies leveraging psychological insights to enhance vaccine acceptance and approaches applying behavioral science to improve immunization policies have been introduced [25,26]. Sentiment analysis of VAERS data is a promising approach to understanding the psychological perceptions of vaccinated patients. This method can potentially improve patient care by addressing knowledge gaps, reducing misconceptions, and fostering better communication and understanding between patients and healthcare providers. This study’s approach demonstrates that sentiment scoring of words is a potentially useful method to describe positive and negative language patterns in VAERS narratives, offering indirect perspectives on reported experiences.

Limitations

The most important limitation of this study is that the VAERS system is designed to collect symptom descriptions rather than direct accounts of patients’ feelings or explanatory narratives. Consequently, our analysis reflects descriptive patterns of vocabulary use, not the actual emotions or lived experiences of vaccine recipients. While the sentiment scores derived from the AFINN lexicon indicate the presence of positive or negative words, they cannot capture the precise meaning intended by the reporter. For example, words such as “resolved” or “strength” received high positive scores, but the database does not specify what was “resolved” or what kind of “strength” was described. These terms may refer to the resolution of symptoms, recovery processes, or other contextual factors, but the exact interpretation cannot be confirmed.

Furthermore, our analysis was limited to identifying and quantifying the occurrence of positive and negative words in free-text narratives, without contextual information about how these terms were used. Thus, the findings should be regarded as descriptive linguistic patterns in symptom narratives, rather than an in-depth exploration of recipients’ emotions. Although some vocabulary identified through sentiment scoring may indirectly reflect psychological or emotional stress, these expressions are embedded within symptom reporting.

Finally, because this study used retrospectively collected data from the VAERS database, strict replicability cannot be ensured. Therefore, the results should be interpreted as descriptive vocabulary patterns identified in the dataset and not as reproducible findings derived from a prospective design.

## Conclusions

This study demonstrates the value of text mining and sentiment analysis in analyzing the vocabulary and identifying positive and negative words used in free-text narratives from the VAERS related to HPV vaccination. Analysis of over 55,000 reports (2009-2023) using the AFINN lexicon revealed that female recipients expressed more negative sentiments than males across all age groups. These findings indicate differences in the use of positive and negative words across sex and age groups. While the present analysis does not allow direct conclusions about psychological or emotional states, such vocabulary patterns may provide useful signals for future research. From a public health perspective, monitoring these patterns could help inform communication strategies and support further studies aimed at improving understanding between patients and healthcare providers.
